# Do Chronic Obstructive Pulmonary Diseases (COPD) Self-Management Interventions Consider Health Literacy and Patient Activation? A Systematic Review

**DOI:** 10.3390/jcm9030646

**Published:** 2020-02-28

**Authors:** Uday Narayan Yadav, Jane Lloyd, Hassan Hosseinzadeh, Kedar Prasad Baral, Mark Fort Harris

**Affiliations:** 1 Centre for Primary Health Care and Equity, UNSW, Sydney NSW-2052, Australia; j.lloyd@unsw.edu.au (J.L.); m.f.harris@unsw.edu.au (M.F.H.); 2 School of Health & Society, University of Wollongong, Wollongong NSW-2522, Australia; hassanh@uow.edu.au; 3 School of Public Health, Patan Academy of Health Sciences, Kathmandu-26500, Nepal; kedarbaral@pahs.edu.np

**Keywords:** Chronic Obstructive Pulmonary Diseases, health literacy, patient activation, self-management

## Abstract

Self-management (SM) includes activities that patients initiate and perform in the interest of controlling their disease and maintaining good health and well-being. This review examines the health literacy and patient activation elements of self-management interventions for Chronic Obstructive Pulmonary Diseases (COPD) patients. We investigated the effects of the intervention on health-related quality of life, self-efficacy, depression, and anxiety among people with COPD. We conducted a systematic review of studies evaluating the efficacy of self-management interventions among COPD patients that also included health literacy or patient activation as keywords. Four electronic databases Medline, EMBASE, PsycINFO, and Google Scholar, were searched to identify eligible studies. These studies were screened against predetermined inclusion criteria. Data were extracted according to the review questions. Twenty-seven studies met the criteria for inclusion. All of the included studies incorporated health literacy components and focused on COPD and self-management skills. Three studies measured health literacy; two showed improvements in disease knowledge, and one reported a significant change in health-related behaviors. Seventeen studies aimed to build and measured self-efficacy, but none measured patient activation. Eleven studies with multicomponent interventions showed an improvement in quality of life. Six studies that focused on specific behavioral changes with frequent counseling and monitoring demonstrated improvement in self-efficacy. Two interventions that used psychosocial counseling and patient empowerment methods showed improvement in anxiety and depression. Most self-management interventions did not measure health literacy or patient activation as an outcome. Successful interventions were multicomponent and comprehensive in addressing self-management. There is a need to evaluate the impact of comprehensive self-management interventions that address and measure both health literacy and patient activation on health outcomes for COPD patients.

## 1. Introduction

Chronic Obstructive Pulmonary Disease (COPD) is a common, preventable, and treatable disease that is characterized by persistent respiratory symptoms and airflow limitation [1]. The treatment and management of COPD is a significant challenge for health systems worldwide [2]. People suffering from COPD often have worsening symptoms, including breathlessness, which requires self-management skills and knowledge. Self-management programs have been demonstrated to slow down the worsening symptoms, prevent exacerbations, and improve quality of life [3]. Self-management behaviors are the practices that patients initiate in the interest of controlling their own disease and maintaining good health and well-being [4]. For COPD, they primarily involve early self-recognition and early self-initiation of treatment for exacerbation, compliance with medication (including immunization), coping with breathlessness, quitting smoking, regular physical exercise, and eating a healthy diet [5]. Systematic reviews suggest that COPD self-management interventions (SMIs) improve health-related quality of life and reduced emergency department visits [6,7,8].

Health literacy (HL) and patient activation (PA) play key roles in self-management interventions. HL helps patients to develop a “skillset” to better manage their health, while PA develops a “mindset” that helps them “to change the lifestyle behavior” [9]. Interventions aimed at using both HL and PA can greatly benefit COPD patients [9]. Health literacy is defined as “the degree to which individuals can obtain, process, and understand the basic health information and services they need to make appropriate health decisions” [8,10] and, “patient activation” refers to the “knowledge, skills and confidence of a person in managing their own health and care” [11].

Addressing health literacy in self-management interventions has been shown to improve individual decisions and actions [12,13] in the area of smoking, nutrition, alcohol, physical activity, and weight control among people with chronic diseases [14]. Similarly, emerging research shows that patient activation improves patient engagement in self-care practices [15] and healthy lifestyle behavior-change programs [16,17]. This review was undertaken to examine health literacy and patient activation in COPD self-management interventions. This systematic review aimed to provide a broad, overarching synthesis of the existing evidence to inform policy, research, and practice in regard to the position of HL and PA in self-management interventions for COPD.

### Research Question

Have COPD self-management interventions included health literacy and patient activation components, and have they measured improvements in health literacy and patient activation?

What are the effects of the intervention on health-related quality of life, self-efficacy, depression, and anxiety among people with COPD in self-management interventions (SMIs)?

## 2. Methods

The methodology was guided by the Preferred Reporting Items for Systematic reviews and Meta-Analyses (PRISMA) [18].

### 2.1. Search Strategy

Four well-known electronic databases, *Medline*, *EMBASE*, *PsycINFO*, and *Google Scholar*, were searched to find eligible RCTs published between 1 January 2008 and 9 December 2019. Combinations of Medical Subject Headings (MeSH) terms, using “OR” and “AND”, were used to operate the electronic databases. To be selected, papers had to have self-management and either health literacy or patient activation search terms, as outlined in the Box 1 below.

Box 1 **List of search terms used in conducting systematic review.**

Self-management: The search terms included any of the following: “Self-management”, “Self-care”, “Self-treat”, “Personalised”, “Self-management Intervention”, “Supporting”, “Engaging” “Pulmonary disease”, “Chronic Obstructive Pulmonary Disease”, and “COPD”.

AND

EITHER

Health literacy: The search terms included any of the following: “Health Literacy”, “Functional health literacy”, “Interactive health literacy”, “Knowledge on health”, “Reading and writing”, “Literacy level”, “Comprehensive health knowledge”, “health information” and “health promotion”, “Chronic Obstructive Pulmonary Disease”, and “COPD”.

OR

Patient activation: The search terms included any of the following: “Patient activation”, “Patient Activation Measure”, “Personalized support”, “Motivation”, Knowledge”, “Skills”, “Confidence”, “Empowerment”, “Chronic Obstructive Pulmonary Disease”, and “COPD”.

The definition of COPD self-management put forward by Effing et al. [19] was used in this review. COPD self-management intervention is defined as being “structured but personalized and often multi-component, with goals of motivating, engaging and supporting the patients to positively adapt their health behavior(s) and develop skills to manage their disease better”.

### 2.2. Inclusion Criteria

Papers should be RCTs or comparative studies in the English language, available in full text, and aimed at the self-management of COPD. Participants had to be diagnosed with COPD. The intervention had to include elements which addressed either HL or PA. Primary outcome measures had to include quality of life (QOL), anxiety and depression, self-efficacy, or the measurement of health literacy and patient activation.

### 2.3. Exclusion Criteria

Conferences abstracts, posters, studies with an intervention period of less than six months, published protocols, studies with patients with cancer as co-morbidity, telehealth interventions, drug trials, and studies reported in other than English language were excluded from this study.

### 2.4. Data Extraction

Initially, one author (U.N.Y.) screened all titles and excluded articles that were irrelevant under the supervision of MFH. After that, five authors (U.N.Y., M.F.H., H.H., J.L. and K.P.B.) used a standardized form based on eligibility criteria to independently review full-text articles in line with the Preferred Reporting Items for Systematic reviews. Finally, MFH separately examined the full-text articles meeting eligibility and exclusion criteria. There were few discrepancies between U.N.Y. and M.F.H. in the excluded data that were resolved in consensus discussion between the two reviewers. A vote of the majority was used to address disagreements during the review of full texts.

## 3. Results

**Figure 1:**
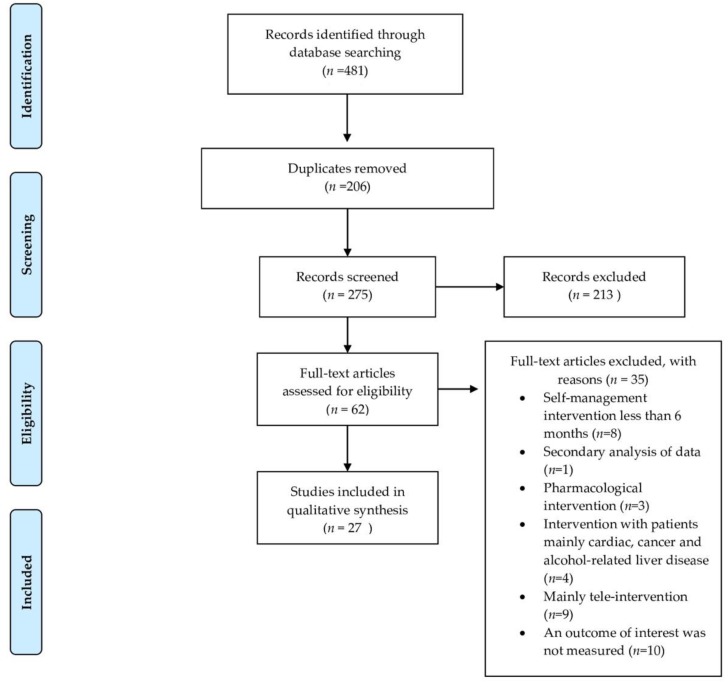
Flow diagram of screening process.

Initially, 481 potentially relevant articles were identified. Twenty-seven studies met the inclusion criteria. Our screening process is depicted in Figure 1.

### 3.1. Health Literacy and Patient-Activation Activities

#### 3.1.1. Health Literacy

All twenty-seven studies included in our review had interventions that address health literacy. This was provided through educational materials (covered information on COPD, self-management skills and COPD medications, breathing techniques, maintaining healthy lifestyle, managing stress and anxiety, inhalation instructions, etc.) and improving clinical communication between health providers and patients. Three [20,21,22] studies specifically measured health literacy in the form of COPD knowledge and self-management skills and behavior. Of these three studies, two [20,22] showed improvements in disease knowledge, and one study [21] reported a significant change in health-related behaviors.

#### 3.1.2. Patient Activation

Eighteen studies [20,22,23,24,25,26,27,28,29,30,31,32,33,34,35,36,37,38] focused on building self-efficacy, but none on building the overall confidence necessary to activate patients to engage in self-management of behaviors. None of the studies in this review included patient activation and measurement.

### 3.2. Outcomes of Self-Management Intervention

#### 3.2.1. Quality of Life

Health-related quality of life was assessed by twenty-five studies [4,7,20,21,22,23,24,25,26,27,28,29,30,31,32,34,35,36,37,38,39,40,41,42,43]. Eleven studies [7,20,22,23,24,25,26,29,31,37,38] reported a significantly higher quality of life in the intervention groups compared to the groups with usual treatment. Of these eleven studies that demonstrated improvement in quality of life, ten studies [20,22,23,24,25,26,29,31,37,38] included activities targeted to HL and PA, while one [7] study used HL but did not report on PA. All eleven studies involved were found to be delivering multicomponent self-management interventions (SMIs) programs, whereby five [20,22,23,31,37] were delivered by multidisciplinary health teams, five by respiratory nurses [7,25,26,29,38], and one [24] by lay tutors. The duration of the interventions varied from six months to twenty-four months. In these studies, health-related quality of life was assessed by using a variety of instruments, including EuroQol-5 Dimension (EQ-5D-3L), SF-36 scale, St.-George’s Respiratory Questionnaire (SGRQ-C), COPD Assessment Test (CAT), Clinical COPD Questionnaire (CCQ), and Short Form Chronic Respiratory Disease Questioner (CRQ-SF).

#### 3.2.2. Self-Efficacy

The effects on self-efficacy were measured in fifteen studies [20,22,23,25,27,29,30,32,33,34,35,38,39,40,41]. Six studies [20,24,27,33,37,44] showed significant improvement in self-efficacy. These involved motivational counselling to encourage participants to set short-term behavioral goals required for self-management of COPD. The interventions were often delivered by health professionals, mainly nurses, physiotherapists, and primary care providers; the exception being the two [24,33] studies that delivered the interventions through trained lay tutors. However, none of these studies provided information on the frequency of the motivational sessions.

#### 3.2.3. Anxiety and Depression

Eleven studies [4,20,24,25,26,31,32,34,36,39,42] measured anxiety and depression as an outcome. Of these, nine [4,20,25,26,32,34,36,39,42] did not show a change in anxiety and depression. Only two interventions [24,31] showed improvement in anxiety and depression scores over the period of the intervention. Of these two interventions, one was delivered by trained lay tutors [24] and another [31] by a primary care team. These studies sought to develop patient empowerment and provided psychosocial counselling.

## 4. Discussion

Despite a wealth of evidence showing beneficial outcomes of COPD self-management programs, substantial gaps remained in the evidence base. To the best of our knowledge, this is the first review to analyze the inclusion of health literacy and patient activation elements in COPD self-management interventions. All the included studies (Table 1) evaluated interventions that aimed to address health literacy to some degree (although HL was not necessarily comprehensively addressed). Three studies [20,21,22] measured disease knowledge as an outcome. None of the authors measured participants’ abilities to read, listen, communicate, and understand the provided information, including health promotion, disease prevention, and the navigation of available services. Although many studies addressed and measured self-efficacy, none specifically developed activities designed to activate patients or measured patient activation as an outcome measure. This suggests the need for their role in self-management for long-term conditions, such as Chronic Obstructive Pulmonary Disease to be further explored and evaluated.

In more than a third of the included studies, self-management interventions improved the quality of life of COPD patients. More importantly, QOL improvements were seen majorly in those interventions that addressed both HL and PA (10 out of 11 interventions that improved QOL) to some degree and offered a comprehensive package of self-management components (individual tailored education sessions on disease and self-management, goal-setting and coping strategies, social support, physical activity, improving confidence, etc.). In line with our findings, other reviews have suggested that multicomponent self-management interventions (SMIs) are significantly effective in improving HRQOL [3,6,45]. However, our finding shows marked variation in the measures of quality of life. This heterogeneity prevented meaningful meta-analysis. Ferrone et al. [46] have suggested the use of a single instrument in future research (i.e., using the Clinical COPD Questionnaire (CCQ)—a 10-item, health-related quality-of-life questionnaire). As reported in another review, we found most studies used generic HRQOL measures (i.e., EQ5D and SF scales), and these reported insignificant differences in quality of life [46]. Overall, our finding shows that multicomponent self-management programs having both HL and PA are more likely to yield promising improvements in QOL.

Of fifteen studies that addressed self-efficacy, only six showed a positive effect on self-efficacy for behavior changes, such as quitting smoking, performing a daily exercise, or taking medicine according to guidelines. Available literature [9] suggests that increased patient engagements with proper confidence-building may help the patients to maintain health behaviors, and this in turn can improve health outcome. The use of interventions which develop patient activation rather than those which focus on specific behaviors (self-efficacy) may be more useful. This may include tailoring approaches to care based on the levels of patient activation. For example, motivational coaching, along with problem-solving skills and social support, targeted for individuals with low activation levels may help them to understand, carry out, and maintain their role in self-managing their conditions over time [9,47]. We found only two studies [24,31] have showed the improvement in depression and anxiety scores. None of the included studies in our review mentioned any defined actions to address depression and anxiety in the SMI. Therefore, this finding of the review should be interpreted with caution.

Self-management programs need to be guided by learning and behavior-change theories that can be tailored to a population’s needs, taking into account literacy, confidence level, ethnic, cultural, and cognitive factors [48]. Interventions aimed at delivering health literacy assume constructing skills for understanding the conditions and relevant information can empower patients, while those aimed at patient activation assume encouragement/motivation standalone can bring positive outcomes [49]. Three of the included studies measured the effect of health literacy, while none measured the effect of patient activation on self-management skills among patients with COPD. Emerging scientific evidence suggests that addressing both health literacy and patient activation components in one intervention might result in better adherence to self-management behaviors in COPD patients [50,51,52]. Motivational and cognitive–behavioral elements and health coaching have been found to be powerful strategies in helping the patient to become a “successful self-manager” [37,53,54]. Thus, the clear understanding between HL and PA, as well as their independent roles and benefits, could help in achieving effective self-management of COPD.

An inherent limitation of this review was the lack of meta-analysis because the intervention and outcome measurements were too heterogeneous. Although multiple databases were searched, using MeSH terms, the search may not have yielded all published relevant studies, given the variation in terminology for “self-management”, “health literacy”, and “patient activation”.

## 5. Conclusions

This review provided insights into how frequently SMI includes features that address health literacy and patient activation. HL interventions were not comprehensive (largely confided to health education) and PA interventions with improving self-efficacy. This suggests the need to further evaluate the impact of comprehensive self-management interventions, which include elements which address both health literacy and patient activation on health outcomes for COPD patients.

## Figures and Tables

**Table 1:** Included studies.

Study ID	Intervention	Participants Characteristics	Follow-Up	Activities Targeted at Health Literacy	Activities Targeted to Activate Patients in any Form	Outcomes	Findings
Chavannes et al. (2009) [23]	Integrated Disease Management delivered by two specialized physiotherapists, a respiratory nurse, a physician assistant, a dietician, a pharmacist, and a supervising primary care physician. **Intervention includes the following:** rapid action plans for exacerbations, personalized physical activity training program (at least three sessions of at least 40 min of physical activity per week over three months) and continuous self-management education including personal goal-setting by motivational interviewing techniques.	Age I/C: 64/63 years Gender (*n* = M/F): 59/67 Setting: outpatients Mean % predicted FEV_1_ I/C = 62 ± 19/66 ± 16 Dyspnea (%) I/C = 36/32 COPD exacerbation *n* (%) I/C = Not reported	One year	Education on disease and self-management skills	Motivational interview	Health-related quality of life (HRQOL)	Improvement in quality of life.
Effing et al. (2011) [39]	Training by physiotherapist **Intervention includes the following:** three-session course (11 h in total) on knowledge about COPD and exercises training for 11 months	Mean age I/C: 62.9 ± 8.1/63.9 ± 7.8 years Gender (*n* = M/F): 89/64 Setting: outpatients Mean % predicted FEV_1_ I/C = 49.6 ± 14.2/50.5 ± 17.0 Mean (SD)dyspnea score I/C = 2.2 ± 1.0/ 2.5 ± 1.1 COPD exacerbation *n* (%) I/C = Not reported	24 months	Self-management sessions	Not described	HRQOL Anxiety and depression	No improvement in the quality of life and in the level of anxiety and depression.
Taylor et al. (2012) [24]	Better Living with Long-term Airways Disease (BELLA) delivered by trained two lay tutors. **Intervention includes the following:** Manualized, a 3 h session once a week, for seven weeks, at a local Community center. A session includes COPD knowledge, maintenance of action plans, skills for self-management and COPD medications and counseling	Mean age I/C: 69.0/70.5 years Gender (*n* = M/F): 78/38 Setting: outpatients Mean % predicted FEV_1_ I/C = 53.9 ± 22.6/54.6 ± 23.4 Mean (SD)dyspnea score I/C = Not reported COPD exacerbation *n* (%) I/C = 60 (77)/26 (68)	Six months	Education on COPD and self-management	Counseling	HRQOL, Health status, self-efficacy, anxiety, depression,	Improvement in QOL (EQ-5D)Improvement in self-efficiency Improvement in anxiety.
Wood-Baker et al. (2012) [25]	Mentoring by Community Health Nurses. **Intervention includes the following:** home visits and telephone coaching, maintenance of patient diary that recorded breathlessness, cough, sputum, wellness, physical activity, and use of reliever medication, along with monthly reflective feedback meetings	Mean age I/C: 66.5 ± 9.5/69.7 ± 9.4 years Gender (*n* = M/F): 46/60 Setting: Inpatients Mean % predicted FEV_1_ I/C = 34.9 ± 14.2/33.8 ± 13.6 Mean (SD) dyspnea score I/C = 3.4 ± 1.1/3.7 ± 1.1 COPD exacerbation *n* (%) I/C = Not reported	12 months	Education on COPD and self-management skills	Mentoring to discuss progress with clients	HRQOL, anxiety and depression, dyspnea and self-efficacy	Improvement in physical functioning of SF-36 scale No improvement in anxiety, depression, dyspnea, and self-efficacy.
Bucknall et al. (2012) [26]	Supported self-management by nurses. **Intervention includes the following:** Participants received four 40-min individual training sessions at home from a study nurse, fortnightly over two months, with further home visits at least every six weeks.	Mean age I/C: 70.0 ± 9.3/68.3 ± 9.2 years Gender (*n* = M/F): 170/294 Setting: outpatients Mean % predicted FEV_1_ I/C = 41.2 ±13.4/39.8±13.8 Mean (SD) dyspnea score I/C = Not reported COPD exacerbation *n* (%) I/C = Not reported	12 months	Education on COPD, respiratory drugs, and self-management skills	Improving patients’ confidence	HRQOL, anxiety and depression, self-efficacy	Clinically relevant improvement in SGRQ No improvement in anxiety and depression and self-efficacy.
Fan Vc et al. (2012) [27]	Intervention delivered by primary care providers **Intervention includes the following:** The Comprehensive Care Management Program (CCMP) included COPD education during four individual sessions and one group session, an action plan for identification and treatment of exacerbations, and scheduled a proactive telephone call for case management.	Mean age I/C: 66.2 ± 8.4/65.8 ± 8.2 years Gender (*n* = M/F): 209/204 Setting: outpatients Mean % predicted FEV_1_ I/C = 38.2 ± 14.3/ 37.8 ± 14.5 Mean (SD) dyspnea score I/C = Not reported COPD exacerbation *n* (%) I/C = Not reported	12 months	Education on COPD, medications, and self-monitoring	Promoting self-monitoring	HRQOL, and self-efficacy	No improvement in health status. Significant improvement in self-efficiency.
Bischoff et al. (2012) [40]	Comprehensive self-management program (CSMP) by practice nurse (two to four sessions scheduled for 4–6 weeks). **Intervention includes the following:** Paper modules on COPD disease knowledge, respiratory drugs, breathing techniques, managing exacerbations, maintaining a healthy lifestyle, managing stress and anxiety (optional), and home exercise (optional).	Mean age self—management, Routine monitoring, and Usual care: 65.5 ± 11.5)/ 65.8 ± 8.3/ 63.5 ± 10.3 years Gender: Male: 37/42/28 Setting: General Practices Mean % predicted FEV_1 for_ self-management, routine monitoring, and usual care = 66.3 ± 16.5/62.9 ± 14.4/67.0 ± 18.0 Mean (SD) dyspnea score = 2.02 ± 0.94/1.87 ± 0.72/1.73 ± 0.76 COPD exacerbation (median, IQ) =1.0 (0–2.0)/1.0 (0–2.0)/0.5 (0–2.0)	18 months	Education on COPD, respiratory drugs, and self-management skills	Not described	HRQOL and Self-efficacy	No improvement in the quality of life. No change in self-efficacy across the group.
Uijen et al. (2012) [28]	Dutch translation of the Canadian COPD-specific self-management program Living Well with COPD delivered by nurses. **Intervention includes the following:** COPD disease knowledge; use of medication and breathing techniques; managing exacerbations; maintaining a healthy lifestyle; managing stress and anxiety; and home exercise. Using motivational interviewing techniques, the practice nurses of each practice gave the program to patients in four individual sessions of 60 min each. Regular monitoring: It includes spirometry, inhalation instructions, and assessment of dyspnea and quality of life.	Mean age usual/ self-management /regular monitoring: 65.3 ± 9.3/ 64.3 ± 11.2/63.5 ± 10.3 years Gender (*n* = M/F): 96/86 Setting: GP patientsMean % predicted FEV_1 for_ usual/self—management, Routine monitoring, and Usual care = 67.0 ± 18.0/65.8 ± 16.3/67.6 ± 15.3 Mean (SD) dyspnea score = not reported COPD exacerbation *n*(%) I/C = not reported	24 months	COPD knowledge and self-management skills	Motivational interviewing	HRQOL	No significant improvement in quality of life.
Casey et al. (2013) [41]	Structured education by nurse and physiotherapists. **Intervention includes the following:** Education sessions.	Mean age I/C: 68.8 ± 10.2 /68.4 ± 10.3 Gender (*n* = M/F): 223/127 Setting: General Practices Mean % predicted FEV_1_ I/C = 57.6 ± 14.3/ 59.7 ± 13.8 Mean (SD) dyspnea score I/C = Not reported COPD exacerbation *n* (%) I/C = Not reported	15 months	Education on COPD and pulmonary rehabilitation	Not described	HRQOL Self-efficacy	No improvement in total. No improvement in self-efficacy.
Mitchell et al. (2014) [20]	Self-management Program for Activity, Coping, and Education (SPACE) delivered by the general practitioner and practice team, including a physiotherapist. **Intervention includes the following:** Disease knowledge, goal setting and coping strategies, exercise regime, and motivational to enhance new lifestyle behaviors.	Mean age I/C: 69 ± 8.0/69 ± 10.1 years Gender (*n* = M/F): 89/97 Setting: outpatients Mean % predicted FEV_1_ I/C = 56.0 ± 16.7/59.6 ± 17.4 Dyspnea grade 2/3/4/5 = (48/24/13/4)/(50/22/14/9) Number of exacerbations in previous 6 months 0/1/2/3/4/o5 = (46/31/7/3/1/1)/(45/33/10/5/1/1)	Six months	Education on COP and self-management	Motivational interviewing	HRQOL, self-efficacy, anxiety, depression	Improvement in quality of life and self-efficiency. No improvement in anxiety and depression. A significant increase in disease knowledge.
Wang et al. (2014) [29]	Health Belief Model Nursing **InterventionIntervention includes the following:**Besides the routine nursing care, a 20- to 30-min HBM based nursing education was implemented for patients in the intervention group every two days after their disease conditions were stable. The tenets of nursing intervention mainly Included the following: (1) assisting the patients to perceive the susceptibility and severity of COPD; (2) assisting them to realize the benefits of the COPD treatment and the initiation of healthy behaviors in COPD; (3) assisting them to conquer the obstacles so that healthy behaviors were applied, and adverse actions were avoided; (4) improving their confidence in managing COPD; and (5) alerting them the signals used to monitor their disease and instructing family members to support patients for the disease management	Mean age I/C: 71.2 (7.4) /71.9(8.1) Gender (*n* = M/F): 50/38 Setting: Inpatients Mean (SD) predicted FEV_1_ I/C = 0.7 ± 0.2/.8±.4 Mean (SD) dyspnea score I/C = 2.4 ± 9/2.3±.8 COPD exacerbation *n* (%) I/C = Not reported	Six months	Education on COPD, medications and self-management skills	Building confidence in managing COPD with assisting patients in perceiving the susceptibility and severity of COPD	HRQOL	Improved mean total scores in the Health Belief Scale, except the perceived disease.
Wilson et al. (2015) [42]	Intervention was delivered by multidisciplinary team. **Intervention includes the following:** 2 h (1 h) individually tailored exercise training and one-hour education program) every three months for one year.	Mean age I/C: 67.3 ± 15.1/69.3 ± 8.9 years Gender (*n* = M/F): 91/57 Setting: outpatients Mean (SD) predicted FEV_1_ I/C = Not reported Mean (SD) dyspnea score I/C = Not reported COPD exacerbation *n* (%) I/C = Not reported	12 months	Education on smoking cessation, healthy eating, and the importance of exercise	Not available	HRQOL Anxiety and/or depression	No improvement in HRQOL. No improvement in anxiety and depression.
Kruis et al. (2014) [30]	Integrated Disease Management (IDM) delivered by General practitioners, practice nurses, and specialized physiotherapists. **Intervention includes the following:** action plans, including early recognition and treatment of exacerbations, encouragement of regular exercise and guideline based physical reactivation, cooperation with secondary care, and instructions in nutritional support.	Mean age I/C: 68.2 ± 11.3/68.4 ± 1.1 years Gender (*n =* M/F): 554/532 Setting: outpatients Mean (SD) predicted FEV_1_ I/C = Not reported Mean (SD) dyspnea score I/C = 2.0 ± 1.3/2.0 ± 1.3 COPD exacerbation *n*(%) I/C = Not reported	24 months	Education on self-management	Motivational interviewing to smoking cessation	HRQOL	No change in HRQOL.
Hernandez et al. (2015) [31]	Intervention delivered by a primary care team (physician, nurse, and social worker) **Intervention includes the following:** 2 h educational program followed by the distribution of patient-specific support material. The intervention consisted of the following: (a) patient’s empowerment for self-management; (b) an individualized care plan; (c) access to a call center; and (d) coordination between the levels of care.	Mean age I/C: 73 ± 8/75 ± 9 years Gender (*n* = M/F): 131/24 Setting: outpatients Predicted % FEV_1_ I/C = 41(19)/44(20)Mean (SD) dyspnea score I/C = 2.7 ± 1.3/2.5 ± 1.3 COPD exacerbation *n* (%) I/C = Not reported	12	Educational training on knowledge of diseases and self-management skills	Patient empower-ment with social support and problem-solving skills.	HRQOL and depression and anxiety	Improved in health-related quality of life. Improvement in anxiety and depression.
Zwerink et al. (2016) [4]	Four weekly self-management meetings supervised by a respiratory nurse and aPhysiotherapist. **Intervention includes the following:** Self-management booklets for patients, patients were trained in completing daily diaries to record major symptoms (breathlessness, sputum production, sputum color) and minor symptoms (cough, wheeze, running nose, sore throat, fever. Patients also were taught to recognize the start of an exacerbation, and to initiate a course of oral prednisolone and/or antibiotics guided by the action plan.	Mean age I/C: 63.1 ± 7.9/63.7 ± 8.0 years Gender (*n* = M/F): 84/50 Setting: outpatients Mean (SD) predicted FEV_1_ I/C = 50.7 ± 16.3/49.6 ± 15.3 Mean (SD) dyspnea score I/C = 2.3 ± 1.06/2.3 ± 1.14 COPD exacerbation *n*(%) I/C = Not reported	24 months	Education on self-management behavior	Not described	HRQOL and, depression and anxiety	No improvement in health-related quality of life. No improvement in anxiety and depression.
Jonsdottir et al. (2015) [32]	Intervention delivered by lung physician and nurses. **Intervention includes the following:** Partnership with people with COPD and their families with patient- family conversation in the presence of a trained nurse, disease information, smoking cessation, and a group meeting.	Mean age I/C: 59.4 ± 4.6/58.6 ± 4.3 years Gender (*n* = M/F): 48/52 Setting: outpatients + GP patients Mean (SD) predicted FEV_1_ I/C = Not reported Mean (SD) dyspnea score I/C = Not reported COPD exacerbation *n* (%) I/C = Not reported	Six months	Education on COPD, Smoking cessation and self-management skills	Motivation on specific behavior like exercise and nutrition	HRQOL, anxiety, and depression	No improvement in QOL and anxiety and depression.
Ko et al. (2017) [7]	Comprehensive care program delivered by respiratory nurses. **Intervention includes the following:** COPD education (1 h) and 3 monthly phone calls to the patients	Mean age I/C: 74.9 ± 7.9/74.6 ± 8.6 Gender (*n* = M/F):172/8 Setting: outpatients Mean (SD) predicted FEV_1_ I/C = 46.7 ± 18.3/44.2 ± 14.7 Mean (SD) dyspnea score I/C = 2 ± 0.8/2.1 ± 0.8 COPD exacerbation *n*(%)I/C = 1.03 ± 1.67/1.38 ± 1.58	12 months	Education on COPD	Not described	HRQOL	Improvement in quality of life.
Poureslami et al. (2016) [33]	Intervention delivered by laypersons and doctors **Intervention includes the following:**Culturally specific educational interventions – two videos (one lay and one clinician video) and one pamphletIn the “lay video,” patients role-played a scenario offering opinions and narratives about COPD self-management in a 12 min video clip	Age: (>75 vs. ≤ 76 years) Gender (*n* = M/F): 71/20 Setting: outpatients Mean (SD) predicted FEV_1_ I/C = Not reported Mean (SD) dyspnea score I/C = Not reported COPD exacerbation *n* (%) I/C = Not reported	Nine months	Educational materials	Developing the confidence to use medication and recognize exacerbations to act correctly)	Self-efficacy	Improvements in the self-efficacy of intervention group participants relative to the control group.
Ng et al. (2017) [44]	Intervention delivered by principal investigator and nurses. **Intervention includes the following:**(1) Self-management education workshops, (2) a patient handbook and (3) a monthly telephone follow-up.	Mean age I/C: Not provided separately Gender (*n* = M/F): Not provided separately Setting: outpatients Mean (SD) predicted FEV_1_ I/C = Not reported Mean (SD) dyspnea score I/C = Not reported COPD exacerbation *n* (%) I/C = Not reported	Six months	Education on COPD, the natural course of the disease, information on how to manage a stable condition, advice on how to prevent complications	Not described	Self-efficacy	Improvement in self-efficacy.
Weldman et al. (2017) [21]	COPD-GRIP intervention delivered by nurses. **Intervention includes the following:** 4 h educational session and animation movie was shown.	Mean age I/C: 68.0 ± 9.6/65.7 ± 9.6 Gender (*n* = M/F): 90/108 Setting: outpatients Mean (SD) predicted FEV_1_ I/C = Not reported Mean (SD) dyspnea score I/C = 2.2 ± 1.3/1.9 ± 1.4 COPD exacerbation *n* (%) I/C = Not reported	12 months	Education Chronic Obstructive Pulmonary Disease – Guidance, Research onIllness Perception)	Not described	HRQOL Health education impact	No significant differences in health-related quality of life. A significant change in health-related behaviors.
Jolly et al. (2018) [34]	Self-management delivered by a nurse. **Intervention includes the following:** Telephone health coaching delivered by a nurse with supporting written documents, a pedometer, and a self-monitoring diary about smoking cessation, physical activity increases, correct Inhaler use technique, and medication adherence.	Mean age I/C: 70.7 ± 8.8/70.2 ± 7.8 Gender (*n* = M/F): 366/211 Setting: outpatients Mean (SD) predicted FEV_1_ I/C = 71.2 ± 18.9/72.1 ± 18.7 Dyspnea *n*(%) I/C = 89 (31)/76 (26) COPD exacerbation *n* (%) I/C = Not reported	12 months	Education on COPD and Self-management behavior	Building patient confidence in identifying an exacerbation early to start rescue drugs	HRQOL, anxiety and Depression Scale, self efficacy	No significant improvement in health-related quality of life, anxiety and depression, and self-efficacy.
Bringsvor et al. (2018) [35]	Two moderators and a registered nurse-delivered intervention and/or physiotherapist. **Intervention includes the following:**A salutogenic orientation was incorporated to improve their self-management capabilities. Sessions covered were: problem-solving, goal setting, symptoms, social challenges, physical activity, nutrition, medication, smoking cessation, exacerbations, and psychological issues.	Mean age I/C: 68.5 ± 8.1/69.3 ± 9.0 Gender (*n* = M/F): 111/71 Setting: Community Mean (SD) predicted FEV_1_ I/C = 45.2 ± 14.4/44.8 ± 16.2 Mean (SD) dyspnea score I/C = 1.8±1.0/1.7 ± 1.1 COPD exacerbation *n*(%) I/C = Not reported	Six months	Information booklet was provided	Salutogenic approach as communi-cation approach	HRQOL, Sel-efficacy	No significant improvement in health-related quality of life and self-efficacy.
Thom et al. (2018) [36]	Intervention delivered by pulmonary nurse and practitioner specialist. **Intervention includes the following:** Health coaching focused on helping patients identify and achieve self-care goals for their COPD using techniques from motivational Interviewing and adult learning models. Specific content included COPD education, action planning for exacerbations, teaching proper inhaler use, and facilitating consultation with a pulmonary nurse practitioner specialist.	Mean age I/C: 60.7 ± 8.0/61.9 ± 7.2 Gender (*n* = M/F):126/66 Setting: urban public health primary care clinics Predicted % FEV_1_ I/C= 0.5 (0.1)/0.6 (0.2) Mean (SD) dyspnea score I/C = 4.3 ± 1.4/4.6 ± 1.4 COPD exacerbation *n* (%) I/C = Not reported	Nine months	COPD education and teaching about the proper use of an inhaler.	Motivational interviewing	HRQOL, depressive symptoms, and self-efficacy	No significant improvement in health-related quality of life, depression, and self-efficacy.
Steurer-Stey et al. (2018) [37]	“Living well with COPD” COPD self-management program based on the Chronic Care Model. **Intervention includes the following:** Program consisted of six group modules, including (1) what is COPD; (2) pharmacological treatment and correct inhalation techniques; (3) breathing techniques and coping strategies aimed at symptom control; (4) how to manage daily activities/energy conservation; (5) the health benefits of physical activity and how to determine barriers and enablers of regular physical activity; and (6) what is an exacerbation and how to prevent, recognize and adequately manage worsening symptoms. Special attention was focused on “red flag” symptoms, like chest pain and/or acute severe dyspnea.	Mean age I/C: 69.3 ± 10.3/67.1 ± 10.0 Gender (*n* = M/F):253/214 Setting: urban public health primary care clinics Mean (SD) predicted FEV_1_ I/C = 52.4 ± 17.6/55.9 ± 16.5 Mean (SD) dyspnea score I/C = 4.6±1.2/4.7 ± 1.6 COPD exacerbation *n*(%) I/C = 51 (71.8)/ 88 (22.2)	Two years	COPD education, teaching about proper use of inhaler and other self-management techniques	Motivational communication and interviewing	HRQOL and self-efficacy	Significant improvement in health-related quality of life and self-efficacy.
Aboumatar et al. (2019) [43]	Intervention was delivered by COPD nurses. **Intervention includes the following:**Individualized COPD self-management support to help patients take medications correctly, recognize exacerbations signs and follow action plan practice breathing exercises and energy conservation techniques, maintain an active lifestyle, seek help as needed, and stop smoking.	Mean age I/C: 63.9 ± 9.6/66.0 ± 10.0 Gender (*n* = M/F): 92/148 Setting: outpatients Mean (SD) predicted FEV_1_ I/C = 35.8 ± 14.2/33.3 ± 16.0 Mean (SD) dyspnea score I/C = Not reported COPD exacerbation *n* (%) I/C = Not reported	Six months	COPD self-management education	Not described	HRQOL	No significant improvement in health-related quality of life.
Lian Hong et al. (2019) [38]	A nurse-led self-management program. **Intervention includes the following:** Every participant received five to six face-to-face, individually tailored education sessions before discharge. The topics included were: (1) what is COPD and what is its impact; (2) respiratory muscle training (pursed-lip breathing and abdominal breathing); (3) medication and appropriate use of inhalation devices; (4) coughing techniques; (5) non-pharmacologic strategies for controlling symptoms; (6) understanding the importance of physical activities for COPD and how to choose the right type of exercise; (7) smoking cessation (if needed); and (8) long-term home oxygen therapy (if needed).	Mean age I/C: 68.7 ± 6.2/69.2 ± 6.1 Gender (*n* = M/F): 121/33 Setting: outpatients Mean (SD) predicted FEV_1_ I/C = 58.4 ± 17.3/59.2 ± 18.2 Mean (SD) dyspnea score I/C = Not reported COPD exacerbation *n* (%) I/C = Not reported	12 months	Individually tailored education sessions	Encourage-ment and reinforcement	HRQOL	A significant improvement in health-related quality of life.
Ferrone et al. (2019) [22]	Intervention was provided by a certified respiratory educator and physician, or usual physician care. **Intervention includes the following:** Case management, education, and skills training, including self-management education.	Mean age I/C: 68.6 ± 9.6/67.9 ± 9.8 Gender (*n* = M/F): 78/90 Setting: outpatients Mean (SD) predicted FEV_1_ I/C = 53.6 ± 14.2/52.0 ± 14.7 Mean (SD) dyspnea score I/C = Not reported COPD exacerbation *n* (%) I/C = 63 (75.0)/63 (75.0)	12 months	Educational sessions and skills training	Patients counseling aimed at developing confidence among patients in various aspects of self-management skills.	HRQOL and Bristol Knowledge question-naire	A significant improvement in health-related quality of life and disease knowledge.

## Abbreviations

CCMP	Comprehensive Care Management Program
COPD	Chronic Obstructive Pulmonary Diseases
DMP	Disease Management Program
C	Control
F	Females
HRQOL	Health-related quality of life
HL	Health Literacy
IDM	Integrated disease management
I	Intervention
M	Males
PSMP	Partnership based Self-management Training
PA	Patient Activation
QOL	Quality of life
SSM	Supported Self-Management
SMI	Self-management Intervention
